# Anti-Angiogenic Properties of BDDPM, a Bromophenol from Marine Red Alga *Rhodomela confervoides*, with Multi Receptor Tyrosine Kinase Inhibition Effects

**DOI:** 10.3390/ijms160613548

**Published:** 2015-06-12

**Authors:** Shuaiyu Wang, Li-Jun Wang, Bo Jiang, Ning Wu, Xiangqian Li, Shaofang Liu, Jiao Luo, Dayong Shi

**Affiliations:** Institute of Oceanology, Chinese Academy of Sciences, Qingdao 266071, China; E-Mails: 12-12sy@163.com (S.W.); wanglijun@qdio.ac.cn (L.-J.W.); beckyjiang0220@163.com (B.J.); wuning@qdio.ac.cn (N.W.); lnu101@163.com (X.L.); lsf909@sina.com (S.L.); luojiao2012@163.com (J.L.)

**Keywords:** bromophenol, anti-angiogenesis, RTKs, multi-target inhibitor, NO, PKB/Akt, eNOS

## Abstract

Bis-(2,3-dibromo-4,5-dihydroxy-phenyl)-methane (BDDPM) is a bromophenol first isolated from *Rhodomelaceae confervoides*. Our previous studies showed that BDDPM exerts PTP1B-inhibiting activity and anti-cancer activity against a wide range of tumor cells while it also showed lower cytotoxicity against normal cells. In the present study, we found that BDDPM exhibits significant activities toward angiogenesis *in vitro*. BDDPM inhibits multiple angiogenesis processes, including endothelial cell sprouting, migration, proliferation, and tube formation. Further kinase assays investigations found that BDDPM is a potent selective, but multi-target, receptor tyrosine kinase (RTKs) inhibitor. BDDPM (10 μM) inhibits the activities of fibroblast growth factor receptor 2 and 3 (FGFR2, 3), vascular endothelial growth factor receptor 2 (VEGFR2) and platelet-derived growth factor receptor α (PDGFRα) (inhibition rate: 57.7%, 78.6%, 78.5% and 71.1%, respectively). Moreover, BDDPM also decreases the phosphorylation of protein kinase B (PKB/Akt) and endothelial nitric oxide synthase (eNOS), as well as nitric oxide (NO) production in a dose dependent manner. These results indicate that BDDPM can be exploited as an anti-angiogenic drug, or as a lead compound for the development of novel multi-target RTKs inhibitors.

## 1. Introduction

Tumor and tumor blood vessels are two major targets in exploiting anti-cancer drugs [1]. More and more cancer patients have benefitted from these two targeted therapeutics, some of the therapeutics are target oncogenic pathways in cancer cells, others are target angiogenic pathways in blood vessels, or both [2]. A rising trend in research demonstrates that drugs that can block more than one molecular target or pathway at the same time can cure cancer more efficiently [3].

Marine algae, an important source of our food supply, have been extensively explored for their use in biological research and medical applications. Bis-(2,3-dibromo-4,5-dihydroxy-phenyl)-methane (BDDPM) is a bromophenol first isolated from the red algae, *Rhodomela larix*. Several studies have reported that BDDPM exerts anti-bacterial and feeding-deterrent effects [4,5,6]. Previously, we synthesized BDDPM and found that it had PTP1B-inhibiting activity [7]. Our recent study also found that BDDPM exerts anti-cancer activity against several cancer cell lines while it has less cytotoxic activity on human umbilical vein endothelial cells (HUVECs) [8]. However, the anti-angiogenic effect of BDDPM has not been reported. Thus, we investigated the effects of BDDPM on angiogenesis and the potential mechanisms *in vitro*.

The process of angiogenesis is highly regulated by multi pro-angiogenic factors, such as vascular endothelial growth factor (VEGF), fibroblast growth factor (FGF), platelet-derived growth factor (PDGF) and epidermal growth factor (EGF) [9]. The intracellular signals are activated when the growth factors binding to their receptors (VEGFR, FGFR, PDGFR and EGFR); then the quiescent endothelial cells activate and begin to sprout, migrate, proliferate, form a tube-like structure and recruit supporting cells for forming a mature vasculature (the normal process of sprouting angiogenesis) [10]. NO, an important mediator, not only functions alone to induce endothelial migration and proliferation in angiogenesis, but also modulates the effects of endothelial progenitor cell and hematopoietic stem cell in vasculogenesis [11]. In endothelial cells, NO is produced by endothelial nitric oxide synthase (eNOS). Pro-angiogenic growth factors bind to their receptors and activate eNOS, upon activation, eNOS catalyzes l-arginine to l-citrulline transformation and NO synthesis [12]. Here, we report that BDDPM inhibits angiogenesis in HUVECs by blocking sprouting, migration, proliferation, and tube formation. The inhibitory effects of BDDPM are potentially mediated through multi-target inhibition against VEGFR2, PDGFRα, FGFR2, FGFR3, and FGFR4; phosphorylation of eNOS and Akt; along with decreased NO production.

## 2. Results

### 2.1. Bis-(2,3-dibromo-4,5-dihydroxy-phenyl)-methane (BDDPM) Inhibits the Proliferation of Human Umbilical Vein Endothelial Cells (HUVECs)

**Figure 1:**
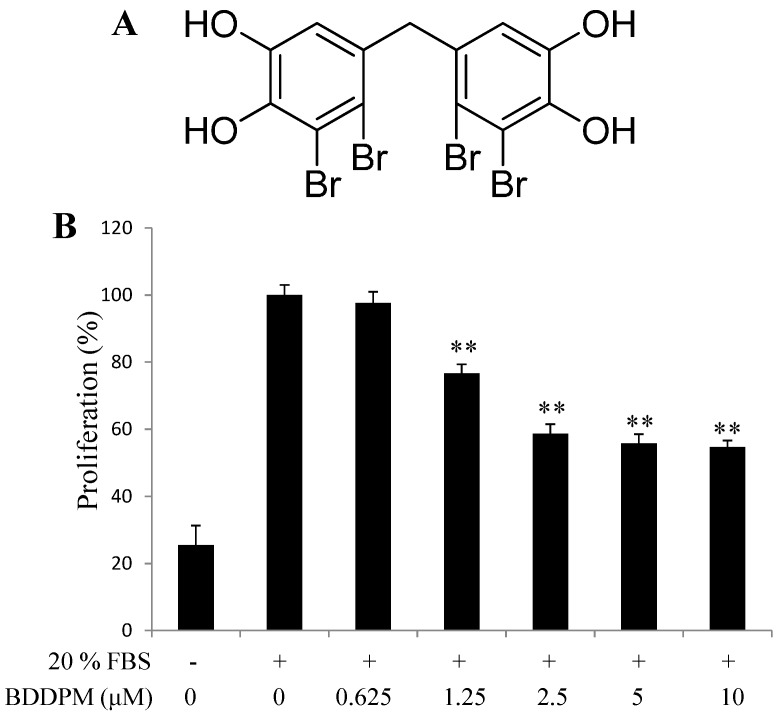
Bis-(2,3-dibromo-4,5-dihydroxy-phenyl)-methane (BDDPM) inhibits human umbilical vein endothelial cells (HUVECs) proliferation. (**A**) Structure of BDDPM; (**B**) The starved HUVECs were stimulated with 20% fetal bovine serum (FBS) and then treated with BDDPM; after 48 h incubation, cell proliferation was evaluated with the CellTiter 96^®^ AQueous One Solution Cell Proliferation Assay. The data shown in the graphs are the mean ± SD values from at least three individual experiments. ** *p* < 0.01 *versus* the control.

To explore the anti-angiogenic effect of BDDPM, we first performed the proliferation assay. The proliferation of HUVECs was significantly stimulated after adding 20% fetal bovine serum (FBS) and 48 h incubation. BDDPM exhibited growth inhibition of HUVECs in a dose-dependent manner, and showed no significant cytotoxicity up to 10 μM compared with the unstimulated control (Figure 1A,B). The IC_50_ value of BDDPM was 3.6 μM, which is much lower than the previous cytotoxic assay (IC_50_, 55.1 μM) [13]. Based on these results, further studies on the anti-angiogenic activity of BDDPM were conducted with 2.5–10 μM.

### 2.2. BDDPM Inhibits HUVECs Migration

**Figure 2:**
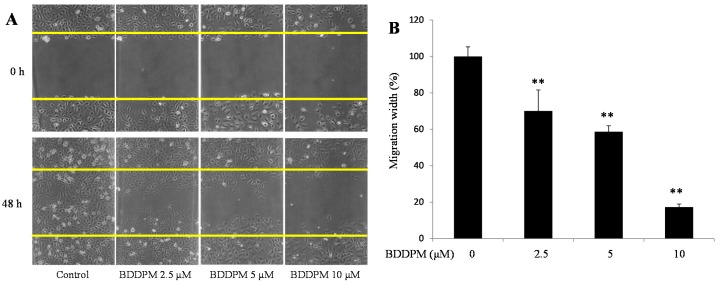
BDDPM inhibits HUVECs migration. After treatment with BDDPM in the presence of 1 μg mitomycin C, HUVECs migration was recorded by microscopy at 0 and 48 h. (**A**) Images of the wounded monolayers of the HUVECs (100× magnification); (**B**) The migrated length of HUVECs. The data shown in the graphs are the mean ± SD values of at least three individual experiments. ** *p* < 0.01 *versus* control.

Endothelial cell migration is another important process in angiogenesis. To investigate the anti-migration effects of BDDPM, a wound healing assay was performed in the presence of 1 μg/mL mitomycin C. As showed in Figure 2A,B, the migration of HUVECs was significantly inhibited by the 48 h treatment with 2.5, 5, and 10 μM of BDDPM.

### 2.3. BDDPM Inhibits Vessel Sprouting in Vitro

**Figure 3:**
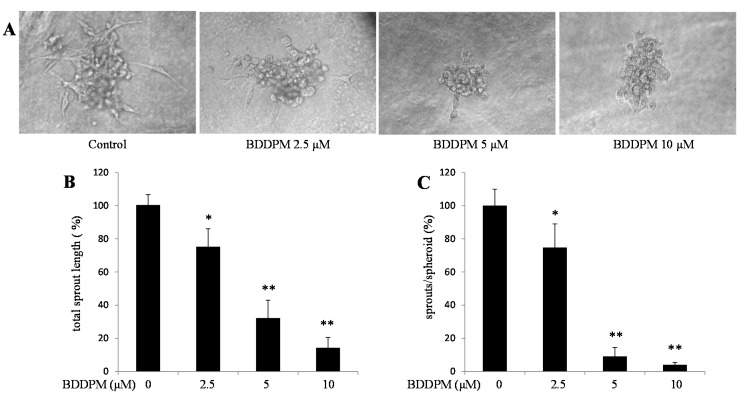
BDDPM inhibits Vessel Sprouting in HUVECs. HUVECs spheroids were seeded in the Matrigel with or without BDDPM. (**A**) After 24 h incubation, the sprouting spheroids were photographed (200× magnification); (**B**,**C**) The number of primary sprouts and the total sprout length were analyzed. The data shown in the graphs are the mean ± SD values of at least three individual experiments. * *p* < 0.05, ** *p* < 0.01 *versus* control.

Next, we used spheroid capillary sprouting assay to study the effect of BDDPM on vessel formation *in vitro*. BDDPM significantly decreased sprout numbers and shortened sprout length during the sprouting of HUVECs (Figure 3A–C).

### 2.4. BDDPM Inhibits Tube Formation on the Matrigel (Growth Factor Enhanced)

**Figure 4:**
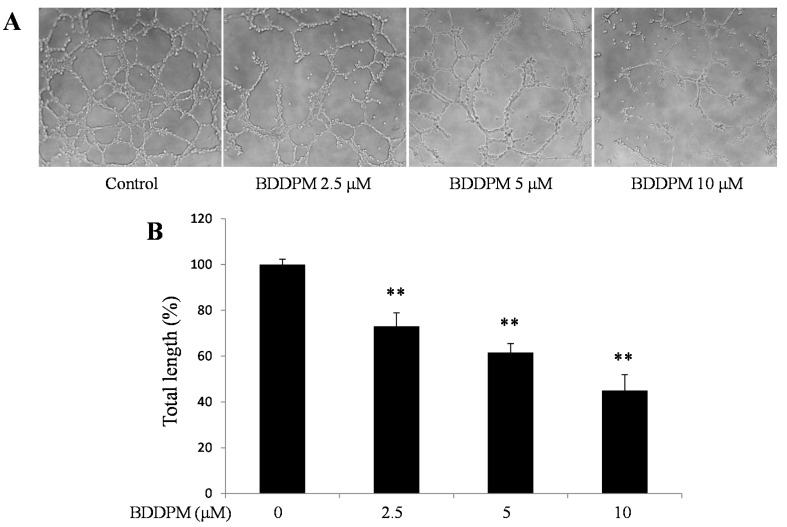
BDDPM inhibits HUVECs tube formation. HUVECs were seeded onto the growth factor enhanced Matrigel-coated 96-well plate with or without BDDPM. (**A**) After 7 h incubation, the tube-like networks were photographed (200× magnification); (**B**) The length of the tube networks was quantified. The data shown in the graphs are the mean ± SD values of at least three individual experiments. ** *p* < 0.01 *versus* control.

To further study the effect of BDDPM on vessel formation *in vitro*, a tube formation assay was performed in the absence or presence of BDDPM on growth factor enhanced Matrigel. As showed in Figure 4A,B, the total length of the endothelial tubes formed on the Matrigel was significantly reduced by BDDPM in a dose-dependent manner.

### 2.5. BDDPM Is a Potent Inhibitor of FGFR2, FGFR3, VEGFR2, and PDGFRα

**Figure 5:**
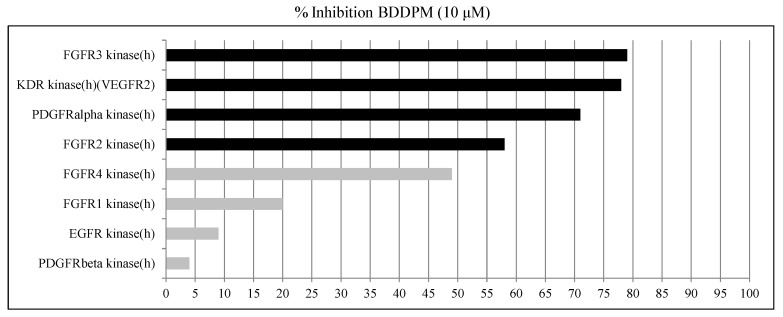
BDDPM is a potent inhibitor of receptor tyrosine kinase. *In vitro* kinase inhibition assays were performed. The inhibition rates were calculated.

To explore the anti-angiogenic mechanism of BDDPM, we set up the kinase inhibition assay. BDDPM (10 μM) potently inhibits the RTKs activities of recombinant FGFR2, FGFR3, VEGFR2 and PDGFRα *in vitro* (inhibition rate: 57.7%, 78.6%, 78.5% and 71.1%, respectively; Figure 5) (Results showing an inhibition higher than 50% are considered to represent significant effects of the test compounds), while displays weak activity against EGFR, FGFR1, PDGFRβ and FGFR4 (inhibition rate: 9.4%, 19.8%, 4.0% and 49.0%, respectively). These results demonstrated that BDDPM is a multi-target inhibitor of FGFR2, FGFR3, VEGFR2 and PDGFRα.

### 2.6. BDDPM Decreases the Phosphorylation of Akt, Endothelial Nitric Oxide Synthase (eNOS) and Inhibits Endothelial Cell NO Production

**Figure 6:**
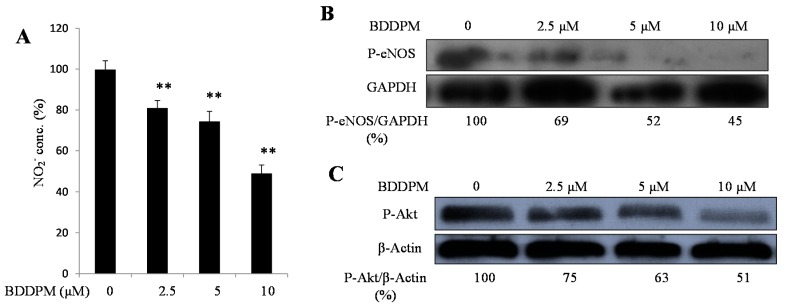
BDDPM decreases NO production and inhibits the phosphorylation of Akt and eNOS. (**A**) HUVECs were incubated with 2.5–10 μM BDDPM for 24 h. Nitrite concentration was determined using the NO Kit; (**B**) HUVECs were incubated with 2.5–10 μM BDDPM. The levels of phosphorylated eNOS or GAPDH were determined by immunoblotting analysis; (**C**) HUVECs were incubated with 2.5–10 μM BDDPM. The levels of phosphorylated Akt or β-Actin were determined by immunoblotting analysis. ** *p* < 0.01 *versus* control.

Finally, we check the downstream signals of angiogenesis to investigate the anti-angiogenic mechanisms of BDDPM. NO, a downstream signal, functions alone or combined with other pro-angiogenic factors during the vessel formation process. We assayed the NO production in the HUVECs. The concentration of NO was significantly reduced after treating the HUVECs with BDDPM for 24 h (Figure 6A).

The phosphorylation of Akt and eNOS are required for NO production. Then, we determined whether BDDPM regulates the phosphorylation of Akt and eNOS by using an immunoblotting assay. BDDPM treatment decreased the phosphorylation of Akt and eNOS in a dose-dependent manner (Figure 6B,C).

## 3. Discussion

It has been reported that bromophenol compounds exhibit a wide spectrum of pharmacological activities including antibacterial, antimicrobial, and antitumor activities [13,14,15,16]. Our recent study found that BDDPM exerts anti-cancer activity against several cancer cell lines [8]. Here, we found that BDDPM inhibits angiogenesis in HUVECs by blocking sprouting, migration, proliferation, and tube formation. BDDPM also functions as a selective but multi-target inhibitor of FGFR2, FGFR3, VEGFR2 and PDGFRα. In addition, BDDPM inhibits the phosphorylation of eNOS and decreases NO production.

Angiogenesis plays an important role in tumor growth, and is an attractive target for anti-cancer drug discovery. The process of angiogenesis is critically regulated by a series of signaling molecules, especially the RTKs (VEGFR, PDGFR, FGFR, and EGFR) [17,18]. Several of them are of interest for drug therapy targets. Most anti-angiogenic drugs targeting RTKs are small molecules selectively inhibiting one of the tyrosine kinases. Some are monoclonal antibodies against growth factors that block the growth factor binding to their receptors [19,20]. Patients with cancer would benefit from the anti-angiogenic therapy by using these RTK small molecule inhibitors or antibodies. But their success is insufficient and even increased metastasis in some models [21,22]. Combinatorial delivery of anti-angiogenic agents or multi-target small molecules may help overcome these challenges. Here, we found that BDDPM functions as a selective, but multi-target, inhibitor of FGFR2, FGFR3, VEGFR2 and PDGFRα. These results indicate that BDDPM could be exploited as a lead compound for the development of novel multi-target RTKs inhibitors.

NO plays a key role in tumor angiogenesis. It can also stimulate cancer cell invasiveness and suppresses host anti-cancer defense [23,24]. It is reported that a NO-blocking agent could reduce tumor angiogenesis and their spontaneous metastases in a mouse model [25]. A single or combined use of NO-blocking agents can be helpful in treating certain cancers [26]. In our study, BDDPM reduced the NO production of HUVECs in a dose-dependent manner. This result suggests that BDDPM influences the RTKs and subsequently affects NO production.

In endothelial cells, NO production is regulated by the eNOS signal pathway. After the growth factors bind to their receptors, the phosphatidylinositol-3-OH-kinase (PtdIns (3) K) activates Akt, and Akt phosphorylates eNOS at serine 1177 [27]. Finally, the phosphorylated eNOS catalyzes l-arginine to l-citrulline and NO [11]. Here, we found that BDDPM decreased the phosphorylation of eNOS that induced by 20% FBS containing medium. This result indicates that NO products inhibition effect of BDDPM might be through the decreased phosphorylation of eNOS. Together with the anti-angiogenic properties of BDDPM, these findings suggest that BDDPM affects the angiogenesis by inhibiting the RTKs and regulating the NO production.

## 4. Experimental Section

### 4.1. Materials

BDDPM was first isolated from *Rhodomelaceae confervoides*, subsequently was synthesized in house (purity, 99%). The synthetic procedures for the preparation of BDDPM have been published in our previously report [7]. Dulbecco’s modified Eagle’s medium (DMEM), fetal bovine serum (FBS), and other cell culture reagents were purchased from Invitrogen (Carlsbad, CA, USA). The high concentration Matrigel was purchased from BD Biosciences (Bedford, MA, USA). The antibody directed against phospho-eNOS (Ser1177) was obtained from BD Biosciences (Bedford, USA). The antibody directed against phospho-Akt (Ser473) was purchased from Cell Signaling Technology (Danvers, MA, USA). The antibody directed against GAPDH was purchased from Abcam Trading Company Ltd. (Shanghai, China). A NO detection kit was purchased from Applygen (Beijing, China). All the other reagents were purchased from Sigma, unless otherwise indicated.

### 4.2. Cell Culture and Proliferation Assay

The HUVECs were obtained from BOSTER, Ltd. (Wuhan, China) and maintained in DMEM supplemented with 10% FBS and 1% antibiotics at 37 °C in an atmosphere containing 5% CO_2_. The cells were split 1:3 when they reached 80% confluence. Cell proliferation was analyzed on exponentially growing cells that were starved for 16 h in 0.2% FBS containing medium and seeded in 96-well microplates (4 × 10^3^ cells per well) as described previously [28]. After exposure to 20% FBS containing medium and/or BDDPM (0.625, 1.25, 2.5, 5, 10 μM) for 48 h, cell proliferation was assessed with the use of the CellTiter 96^®^ AQueous One Solution Cell Proliferation Assay (Promega, Madison, Wisconsin, WI, USA) according to manufacturer’s instructions.

### 4.3. Wound Healing Assay

As described previously [29], the exponentially growing HUVECs (2.5 × 10^5^ cells per well) were cultured in 6-well plates and starved overnight in 2% FBS medium until they reached 90% confluence. A single wound was then scratched in the center of the cell monolayers with a 200 μL sterile plastic pipette tip. The wounded monolayers were washed twice with 1× PBS to remove the non-adherent cells and were incubated with various concentrations of BDDPM for 48 h in the presence of 1 μg/mL of mitomycin C (for mitotic inactivation). To measure the length of the endothelial cells that had migrated from the edge of the injured monolayer, images were obtained immediately after wounding and after a 48 h incubation period, using a phase-contrast microscope (Olympus, Tokyo, Japan). The length was measured by the Image Pro Plus v 6.0 software (Media Cybernetics, Inc., Bethesda, MD, USA).

### 4.4. Spheroid Capillary Sprouting Assay

Sprouting analysis was performed as described previously with some modifications [28]. Briefly, HUVECs (1 × 10^3^ cells) were incubated overnight in hanging drops in DMEM medium containing 20% methylcellulose (Sigma-Aldrich, Bornem, Belgium) to form spheroids. For mitotic inactivation, Mitomycin C (500 ng/mL) was added to this medium. Spheroids were then embedded in high concentration Matrigel and 20% FBS containing DMEM medium mixture (*v*/*v*: 1:2) with compound and vehicle. After 1 h incubation at 37 °C, the gel was covered with 20% FBS containing medium in the presence of BDDPM and vehicle and cultured for 24 h to induce sprouting. Analysis of the number of primary sprouts and the total sprout length (cumulative length of primary sprouts and branches per spheroid) was done using the Image Pro Plus v 6.0 software.

### 4.5. Capillary-Like Tube Formation Assay

As described previously [29], high concentration Matrigel was added to a 96-well plate (50 μL per well) and allowed to polymerize for 1 h at 37 °C. The HUVECs (5.5 × 10^4^ cells per well, 200 μL per well) with or without BDDPM, were seeded onto the surface of the Matrigel. After a 7 h incubation period, cellular morphological changes and tubular structure formation were observed under a phase-contrast microscope (Olympus). The images were captured and the degree of tube formation was quantified by measuring the lengths of the tubes using the Image Pro Plus v 6.0 software.

### 4.6. In Vitro Kinase Inhibition Assays

The *in vitro* kinase inhibition protocols have been described previously: EGFR [30], FGFR1 [31], FGFR2 [32], FGFR3 and FGFR4 [33], KDR (VEGFR2) [34], PDGFRα [35] and PDGFRβ [36]. The ATP concentration was 100 nM in the assays for EGFR, FGFR1, FGFR3, FGFR4, KDR (VEGFR2) and PDGFRα, and 25 nM in the assays for PDGFRβ and FGFR2. The incubation time was 60 min in the assays for FGFR1, FGFR4, KDR (VEGFR2) and PDGFRα, 15 min in the assays for EGFR and FGFR2, and 90 min in the assay for FGFR3. The results expressed as a percent inhibition of control-specific activity. Results showing inhibition higher than 50% were considered to represent significant effects of the test compound.

% Inhibition = 100 − (measured specific activity)/(control specific activity) × 100
(1)

### 4.7. Immunoblotting Analysis

Immunoblotting analysis was performed as described previously [29]. Briefly, the HUVECs (2 × 10^5^ cells per well) were cultured in 6-well plates. When they reached 80% confluence, the cells were serum starved for 16 h in 2% FBS containing medium and pretreated with BDDPM for 30 min. Subsequently, the cells were stimulated with 20% FBS containing medium for another 30 min and washed with ice-cold PBS and lysed with lysis buffer (50 mM Tris-HCl, 1% Triton X-100, 0.5% sodium deoxycholate, 150 mM NaCl, 1 mM EDTA, 1 mM phenylmethylsulfonyl fluoride (PMSF), 1-mM sodium orthovanadate, 1 mM NaF, and 0.2% protease inhibitor cocktail; pH 7.2). Proteins were separated by sodium dodecyl sulfate-polyacrylamide gel electrophoresis and were subsequently transferred to nitrocellulose membranes. The membranes were blocked with 5% skim milk in 1× Tris-buffered saline containing 0.1% Tween 20 (TBST) for 1 h at room temperature and were then incubated overnight at 4 °C with a primary antibody. The following day, the membranes were washed with TBST and were probed with a secondary antibody. The bands were detected using enhanced chemiluminescence reagents (Thermo Fisher Scientific Inc., Shanghai, China).

### 4.8. NO Measurement

One means to investigate nitric oxide formation is to measure nitrite (NO_2_^−^), which is one of two primary, stable and nonvolatile breakdown products of NO. The NO levels in the HUVECs were measured with the NO_2_^−^ detection kit. Briefly, the HUVECs were cultured in 24 wells plates (1 × 10^5^ cells per well). After overnight incubation, the cells were starved for 16 h in 2% FBS containing medium. Then, the cells were exposed to 20% FBS containing medium with or without BDDPM. Twenty-four hours later, the supernatant was collected, and NO production was determined following the protocol supplied with the kit (Applygen, Beijing, China).

### 4.9. Statistical Analysis

All the experiments were performed at least three times, and the data are presented as mean ± SD values. Differences between the mean values were assessed using one-way analysis of variance. For all the analyses, *p* < 0.05 was considered significant. Statistical analyses were performed using SPSS 17.0 (SPSS, Inc., Chicago, IL, USA).

## 5. Conclusions

In summary, BDDPM exhibits significant activities toward angiogenesis *in vitro*, including endothelial cell proliferation, migration, tube formation, and sprouting. BDDPM is a potent multi-target RTKs inhibitor, which inhibits the activities of FGFR2, FGFR3, VEGFR2 and PDGFRα. BDDPM also decreases the phosphorylation of Akt-eNOS and reduces the production of NO, the downstream signals of angiogenesis. These results indicate that BDDPM could be exploited as a multi-target anti-angiogenic drug, or as a lead compound for the development of novel multi-target RTKs inhibitors.
